# The functional variant rs34330 of *CDKN1B* is associated with risk of neuroblastoma

**DOI:** 10.1111/jcmm.13226

**Published:** 2017-06-30

**Authors:** Mario Capasso, Lee D. McDaniel, Flora Cimmino, Andrea Cirino, Daniela Formicola, Mike R. Russell, Pichai Raman, Kristina A. Cole, Sharon J. Diskin

**Affiliations:** ^1^ Dipartimento di Medicina Molecolare e Biotecnologie Mediche Università degli Studi di Napoli Federico II Naples Italy; ^2^ Istituto di Ricerca Diagnostica e Nucleare IRCCS SDN Naples Italy; ^3^ Division of Oncology Children's Hospital of Philadelphia Philadelphia PA USA; ^4^ Center for Childhood Cancer Research Children's Hospital of Philadelphia Philadelphia PA USA; ^5^ CEINGE Biotecnologie Avanzate Naples Italy; ^6^ Department of Pediatrics Perelman School of Medicine University of Pennsylvania Philadelphia PA USA; ^7^ Abramson Family Cancer Research Institute Perelman School of Medicine at the University of Pennsylvania Philadelphia PA USA

**Keywords:** Neuroblastoma, SNP, *CDKN1B*, genetic association, GWAS, rs34330

## Abstract

The genetic aetiology of sporadic neuroblastoma is still largely unknown. We have identified diverse neuroblastoma susceptibility loci by genomewide association studies (GWASs); however, additional SNPs that likely contribute to neuroblastoma susceptibility prompted this investigation for identification of additional variants that are likely hidden among signals discarded by the multiple testing corrections used in the analysis of genomewide data. There is evidence suggesting the *CDKN1B*, coding for the cycle inhibitor p27Kip1, is involved in neuroblastoma. We thus assess whether genetic variants of *CDKN1B* are associated with neuroblastoma. We imputed all possible genotypes across *CDKN1B* locus on a discovery case series of 2101 neuroblastoma patients and 4202 genetically matched controls of European ancestry. The most significantly associated rs34330 was analysed in an independent Italian cohort of 311 cases and 709 controls. *In vitro* functional analysis was carried out in HEK293T and in neuroblastoma cell line SHEP‐2, both transfected with pGL3‐CDKN1B‐CC or pGL3‐CDKN1B‐TT constructs. We identified an association of the rs34330 T allele (‐79C/T) with the neuroblastoma risk (P_combined_ = 0.002; OR = 1.17). The risk allele (T) of this single nucleotide polymorphism led to a lower transcription rate in cells transfected with a luciferase reporter driven by the polymorphic p27Kip1 promoter (*P* < 0.05). Three independent sets of neuroblastoma tumours carrying ‐79TT genotype showed a tendency towards lower *CDKN1B* mRNA levels. Our study shows that a functional variant, associated with a reduced *CDKN1B* gene transcription, influences neuroblastoma susceptibility.

## Introduction

Neuroblastoma, an embryonal tumour arising in tissues of the sympathetic nervous system, is the most common cancer diagnosed during the first year of life and accounts for 15% of all deaths due to childhood malignancies 1. Familial neuroblastoma is rare (approximately 1%), and most of these cases harbour germline mutations in *ALK* 1. In contrast, the genetic bases of sporadic neuroblastoma remain largely unknown 2, 3. Through a genomewide association study (GWAS) of sporadic neuroblastoma, we have reported common single nucleotide polymorphisms (SNPs) associated with neuroblastoma susceptibility at *CASC15* 4, *BARD1* 5, *LMO1* 6, *DUSP12* 7, *HSD17B12* 7, *DDX4/IL31RA* 7, *HACE1* 8 and *LIN28B* 8 loci. Importantly, many of these GWAS‐identified genes have been shown to be key factors in both initiating and sustaining tumorigenesis 6, 8, 9, 10, 11. These results suggest that neuroblastoma tumorigenesis could be the result of multiple genetic alterations and that these variants can also influence the clinical outcome of disease. Despite these notable achievements, additional predisposing variants remain to be discovered as the identified risk variants only explain a small proportion of neuroblastoma heritability. Additional genetic risk variants are likely hidden among signals discarded by the multiple testing correction required in the analysis of GWAS data. Different research strategies have been proposed for extracting true‐positive loci such as meta‐analysis and genotype imputation of GWAS data sets 12. In two recent GWAS follow‐up studies based on two different approaches incorporating prior knowledge, we have discovered additional neuroblastoma susceptibility loci within *NEFL* and *TP53* 13, 14.

The *CDKN1B* gene encodes p27kip1, an inhibitor of cyclin/cyclin‐dependent kinase complexes 15 which are crucial for cell cycle progression and development. Loss of expression of p27kip1 has been described as a frequent event in several human cancers 16 conferring a proliferative advantage that can lead to tumour formation. This behaviour suggests p27 may function as a tumour suppressor, although it is rarely mutated in cancer 17, 18. Diverse lines of evidence from *in vitro* to clinical studies suggest that *CDKN1B* can play a role in neuroblastoma development and progression. The regulation of p27 levels has been implicated in the control of proliferation of neuroblastoma cells; indeed, the retinoic acid strongly increases p27Kip1 levels by down‐regulating the ubiquitin–proteasome p27Kip1 degrading pathway in neuroblastoma cell lines 19. Wallick *et al*. showed that α‐difluoromethyl‐ornithine (DFMO) and S‐adenosylmethionine decarboxylase inhibitor (SAM486) treatments induce significant accumulation of the p27kip1 protein and cause p27kip1/Rb‐coupled G1 cell cycle arrest in *MYCN*‐amplified neuroblastoma tumour cells 20. Finally, low expression of p27kip1 has been found as prognostic marker independently from *MYCN* amplification in human neuroblastoma 21, 22.

Because somatic mutations of the *CDKN1B* gene are rare in human malignancies 17, 18 and the reduced protein and gene expression of *CDKN1B* is frequently observed in neuroblastoma 21, 22, it has been suggested that common genetic variants in *CDKN1B* may be associated with reduced mRNA and protein expression and increased risk of neuroblastoma. To identify genetic variants associated with neuroblastoma at *CDKN1B* locus, a large cohort of 2101 patients and 4202 controls was used as a discovery set to perform genotype imputation and genetic association analyses, and an independent population of 311 cases and 709 controls was used as replication set. Here, we show that the ‐79 C/T (rs34330) polymorphism acts as a genetic risk factor for neuroblastoma by affecting the transcription rate, as indicated by the functional assays performed in this study.

## Materials and methods

### Study population

This study included a GWAS data set of 2101 neuroblastoma patients registered through the North American‐based Children's Oncology Group (COG) and 4202 cancer‐free children of self‐reported Caucasian ancestry who were recruited and genotyped by the Center for Applied Genomics at the Children's Hospital of Philadelphia (CHOP). European‐American cases and controls have been described in detail in a previous publication 8. In addition, this study also included 311 neuroblastoma patients and 709 cancer‐free controls of Italian origin. Additional details and eligibility criteria for genotyping of both populations are reported in Data S1. This study was approved by the Ethics Committee of the Medical University of Naples and the CHOP.

### SNP genotyping

The European‐American DNA samples were genotyped using the Illumina Infinium II BeadChip HumanHap550 v1, v3 and Quad610 arrays according to methods detailed elsewhere 8. The SNP rs34330 (*CDKN1B*) was analysed in the Italian cohort. The DNA samples were genotyped using SNP Genotyping Assay on 7900HT Real‐Time PCR System (Applied Biosystem, ABI Inc., CA, USA). More details on SNP genotyping are reported in Data S1.

### Genotype imputation and genetic association analysis of *CDKN1B* locus

Imputation analysis was performed on 2101 cases and 4202 controls of European ancestry. Prephasing was performed first using SHAPEIT 23, followed by imputation using 1000 Genomes data (Phase I Release 3) and IMPUTE2 24. SNPs with minor allele frequency (MAF) <1% and/or IMPUTE2‐info quality score <0.8 were removed. To account for imputation uncertainty, the remaining SNPs were tested for association with neuroblastoma using the frequentist test under the additive model with the ‘score’ method implemented in SNPTEST 25. Additional details are reported in Data S1.

### SNP–gene expression correlation analysis in tumour tissue and neuroblastoma cell lines

Two genomewide mRNA expression data sets (GSE3960 and phs000467) with corresponding SNP genotype data (pha002845 and phs000467) were analysed to assess SNP‐gene expression correlation at the *CDKN1B* locus in 51 and 130 neuroblastoma patients, respectively. SNP–gene expression correlation was further evaluated in a panel of 20 neuroblastoma cell lines (GSE78061). Additional details are reported in the Data S1. These neuroblastoma cell lines were authenticated by Nucleic Acid/PCR Core (NAPCore) facility at the Joseph Stokes Jr Research Institute by short‐tandem‐repeat (STR) DNA fingerprinting with an AmpFLSTR Identifiler PCR Amplification kit (Applied Biosystem), according to the manufacturer's recommendations. Last re‐authentication of the cells was performed on 01/05/15.

### *In vitro* functional analysis

The human HEK293T and SHEP‐2 cell lines were obtained from the American Type Culture Collection. HEK293T cell lines were grown in Dulbecco's modified Eagle's medium (DMEM Sigma‐Aldrich, Budapest, Hungary); SHEP‐2 were cultured in RPMI; both media were supplemented with 10% heat‐inactivated foetal bovine serum (Sigma‐Aldrich), 1 mM L‐glutamine, penicillin (100 U/ml) and streptomycin (100 μg/ml; Invitrogen, Carlsbad, CA, USA); and the cells were cultured at 37°C, 5% CO2 in a humidified atmosphere. The cumulative culture length of the cells was less than 6 months after resuscitation. Early‐passage cells were used for all experiments, and they were not re‐authenticated. HEK293T and SHEP‐2 were transfected with pGL3‐CDKN1B‐CC or pGL3‐CDKN1B TT constructs and were subsequently starved in serum‐free medium for 8 hrs. Cells were induced to re‐enter the cell cycle by the addition of fresh medium supplemented with 10% FCS for 0, 12 and 24 hrs. At these time‐points, the cells were harvested, lysed and analysed for luciferase activity. Luciferase activity was normalized to the internal Renilla control, after subtraction of pGL3 Basic Vector activity. Data represent the means ± S.D. of three independent transfections.

### Gene expression data for survival analysis and association with neuroblastoma stages

*CDKN1B* normalized gene expression array data of two independent sets of neuroblastoma patients were downloaded from the website R2: Genomics Analysis and Visualization Platform (http://r2.amc.nl): (*i*) composed of 498 samples (4 × 44K Agilent; GSE49710); and (*ii*) composed of 88 samples (Affymetrix Human Genome U133 Plus 2.0 Array; GSE16476).

### Statistical analysis

The deviation of the SNP genotypes from the Hardy–Weinberg equilibrium was evaluated using the goodness‐of‐fit chi‐square test in controls. For genotyped SNPs, two‐sided chi‐square tests were used to evaluate differences in the distributions of allele frequencies between all patients and controls. ORs and 95% confidence interval (CI) were also calculated. To test association of gene expression levels with overall survival, individual gene expression profiles were dichotomized by median split into ‘high’ or ‘low’ expression groups, and Kaplan–Meier survival curves were plotted for each group. Mann–Whitney *U*‐test was used to compare the differences in the mRNA expression levels. For the SNP meta‐analysis, a fixed‐effect model, Cochran's *Q*‐test and *I* ^*2*^ test for heterogeneity were performed as implemented in PLINK (http://pngu.mgh.harvard.edu/~purcell/plink/). For the combined analysis of *P*‐values referring to the SNP–gene expression, the Fisher method as implemented in MetaP (http://people.genome.duke.edu/~dg48/metap.php) was performed.

## Results

### Association between clinical outcome and *CDKN1B* expression

**Figure 1:**
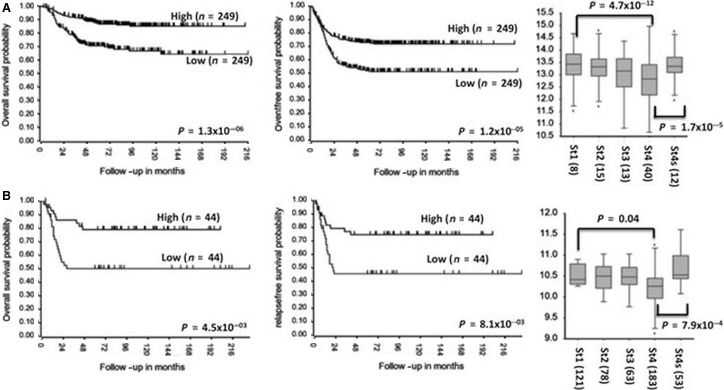
*CDKN1B* expression is associated with good outcome and favourable neuroblastoma stages. (**A** and **B**) Kaplan–Meier analysis using published array data from two independent sets of tumours [R2: Genomics Analysis and Visualization Platform (http://r2.amc.nl)] and changes in expression for *CDKN1B* in favourable stage using published array data.

To verify a potential role of *CDKN1B* in neuroblastoma, we evaluated whether its expression is associated with clinical outcome of patients. Analysis of two publicly available gene expression array data of neuroblastoma showed that high *CDKN1B* expression is associated with better overall and event‐free survival and favourable stages (Fig. 1A and B). The low *CDKN1B* expression in advanced‐stage neuroblastoma suggests this gene may play a role in the neuroblastoma disease process 21, 22.

### Association between the SNP rs34330 ‐79 C/T and neuroblastoma susceptibility

To discover SNPs associated with neuroblastoma at the *CDKN1B* locus (Table 1), we performed a genotype imputation on a discovery case series of 2101 neuroblastoma patients and 4202 genetically matched controls of European ancestry. Within the 1‐kb region surrounding *CDKN1B*, six SNPs resulted to be nominal significant (*P* ≤ 0.05). Interestingly, the T allele of rs34330, which maps to the *CDKN1B* promoter region (‐79 C/T), has been reported to be associated with other tumours including breast 26, endometrial 27, lung 28 and thyroid cancer and with low gene expression of *CDKN1B* 29. Together, these data led us to exclude other SNPs from further analyses, and to focus our attention on rs34330 (*P* = 0.015; OR = 1.15).

**Table 1:** SNP associations at CDKN1B locus (±1Kb) in the discovery cohort of European ancestry

SNP	pos	Role	A1	A2	F_A	F_U	*P*	OR	L95	U95
achr12:12869553:I	12869553		T	TG	0.105	0.103	0.834	1.01	0.90	1.14
ars248743	12869787	5′ upstream	T	C	0.037	0.044	**0.028**	0.82	0.68	1.00
ars36228499	12869936	5′ upstream	A	C	0.439	0.446	0.856	0.97	0.90	1.05
ars36228498	12869972	5′ upstream	G	T	0.156	0.167	**0.038**	0.92	0.83	1.02
rs34330	12870695	5′ UTR	T	C	0.259	0.233	**0.015**	1.15	1.06	1.25
rs2066827	12871099	Coding	G	T	0.226	0.246	**0.021**	0.89	0.82	0.98
achr12:12872484:I	12872484		C	CA	0.300	0.274	**0.003**	1.14	1.05	1.23
ars34329	12873233	Intronic	G	C	0.291	0.304	0.071	0.94	0.87	1.02
rs3093736	12873301	Intronic	A	G	0.032	0.033	0.470	0.96	0.78	1.19
ars34328	12873653	Intronic	T	C	0.407	0.420	0.282	0.95	0.88	1.02
ars34327	12873748	Intronic	T	C	0.492	0.467	**0.010**	1.11	1.03	1.19
ars7330	12874917	3′ UTR	C	A	0.378	0.378	0.701	1.00	0.93	1.08
ars1420023	12876111	3′ downstream	G	C	0.120	0.116	0.454	1.04	0.93	1.17

A1, minor allele; A2, major allele; F_A, minor allele frequency in cases; F_U, minor allele frequency in controls; L95, lower bound of 95% CI for OR; U95, upper bound of 95% CI for OR; pos: position based on hg19.

a Imputed SNPs in the discovery cohort of European ancestry.

The nominal significant SNPs are in bold.

### Replication study for the SNP rs34330

We genotyped rs34330 in 311 cases and 709 controls of Italian origin and tested for association with neuroblastoma. We confirmed the association between the T allele and risk of neuroblastoma (T allele frequency in cases 0.25 and controls 0.21, *P* = 0.04, OR = 1.25, 95% CI = 1.00–1.56). A meta‐analysis of the two studies showed a combined *P*‐value of 0.002 (*Q* statistic *P* = 0.35, *I* ^*2*^= 0%, OR = 1.17). No significant correlation was observed in either the discovery or replication cohort between rs34330 genotype and the following clinical covariates: risk group, INSS stage, MYCN status and age at diagnosis.

### Correlation analysis between the rs34330 and gene expression

**Figure 2:**
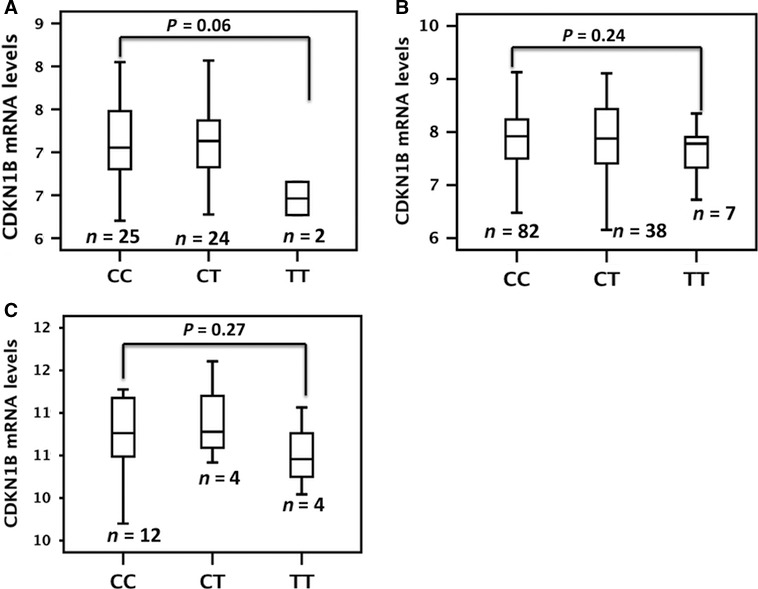
*CDKN1B* genotype and gene expression association. Microarray‐based expression profiling on two independent sets of primary tumours (**A** and **B**) and neuroblastoma cell lines (**C**) shows a trend towards the association between *CDKN1B* expression and rs34330.

**Figure 3:**
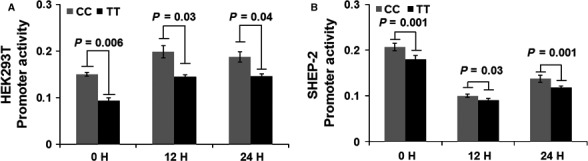
The T allele of rs34330 down‐regulates promoter activity. Transcriptional activity of the pGL3‐CDKN1B‐CC (CC) and pGL3‐CDKN1B‐TT (TT) constructs in (**A**) HEK293T and (**B**) SHEP‐2 neuroblastoma cells.

To investigate whether rs34330 has a *cis*‐effect on *CDKN1B*, we tested for SNP–gene expression associations. The analysis of gene expression variation using genomewide expression and SNP arrays of neuroblastoma tumours demonstrated that the SNP might affect expression of *CDKN1B*. In particular, the presence of the risk allele T correlated with decreased *CDKN1B* mRNA expression in a set of 51 tumours (Fig. 2A; *P* = 0.06). A trend towards association between low mRNA levels and the presence of the risk allele T was observed without reaching the threshold for statistical significance in an analysis including 130 tumours from patients (Fig. 2B; *P* = 0.24) and 20 from neuroblastoma cell lines (Fig. 2C; *P* = 0.27). Notably, after meta‐analysis of the three single *P*‐values, we observed a further decrease in the significance level (combined *P* = 0.08). This strengthens the correlation between the SNP rs34330 and *CDKN1B* expression. Finally, induction of promoter activity of the construct containing rs34330‐T alleles was lower than that of the construct containing C alleles as assessed by luciferase report gene assay in neuroblastoma SHEP‐2 cells and in HEK293T cells (Fig. 3).

## Discussion

In the present study, we have analysed a total of 2412 neuroblastoma cases and 4911 controls and demonstrate that the SNP rs34330, located in the promoter of *CDKN1B* gene, is associated with neuroblastoma. In particular, the minor allele T confers risk for neuroblastoma development and correlates with decreased mRNA levels of *CDKN1B*. We also show that high *CDKN1B* mRNA expression levels correlate with favourable tumour stages and show association with a better clinical outcome. Accordingly, different studies suggest that *CDKN1B* may promote neural differentiation, in addition to regulating the cell cycle. Indeed, it has been demonstrated that cell cycle exit and neural differentiation are strongly regulated by p27kip1 and cyclin D/CDK complexes 30. Moreover, p27kip1 appears to induce neuronal differentiation through other mechanisms. Indeed, different studies suggest that p27kip1 can participate in neural differentiation as a cytosolic mediator of (*i*) neural differentiation through stabilization of helix‐loop‐helix‐type transcription factor neurogenin 31 and (*ii*) microtubule polymerization 32.

Recent literature data suggest *CDKN1B* plays a role in neuroblastoma. Indeed, p27kip is involved in GRP (gastrin‐releasing peptide)/GRP‐R pathway, whose inhibition increases p27kip expression levels in neuroblastoma cells 33. The same pathway amplifies the expression and accumulation of *PTEN* in the cytoplasm of cells suggesting that p27 promotes the function of *PTEN* as a critical tumour suppressor in tumorigenesis 33. The low expression of p27kip1 is associated with unfavourable neuroblastomas independently from *MYCN* amplification 21, 22. The acid retinoic treatment of neuroblastoma cell lines induces growth arrest by reducing the rate of p27kip1 degradation 19. Therefore, the evidence‐based literature supports our hypothesis that functional DNA variants in the promoter region of *CDKN1B* can influence neuroblastoma susceptibility and that low expression of *CDKN1B* plays a role in malignant neuroblastic transformation and disease progression by altering different cell functions such as the normal neuronal differentiation programme or cell cycle regulation.

The association of the SNP rs34330 has been found in diverse tumours 26, 27, 28. Particularly, Landa *et al*. showed that TT genotype of rs34330 is associated with thyroid cancer and correlated with low‐expression level p27kip1 protein 29. However, other genetic variants of *CDKN1B* have been associated with other tumours such as prostate cancer 34, oral squamous cell carcinoma 35, invasive epithelial ovarian cancer 36, high‐grade breast tumour 37 and lymph node metastasis in breast cancer 38. Together, these data suggest that the misregulation of p27kip protein, due to the genetic variants, can affect tumour suppressor function of *CDKN1B*. Therefore, the dysfunction of p27kip may lead to cancer development and/or may affect the outcome of anticancer therapies. Recent studies demonstrate that anticancer drugs can modulate the p27 expression 39, 40.

One limitation of this research work is that the risk SNP was nominally significantly associated with disease (*P* < 0.05). Thus, genetic replication efforts are needed in populations with different ethnic origins to further validate this genetic association.

In conclusion, this study has demonstrated that hypothesis‐driven GWAS follow‐up study is a useful strategy for identifying novel disease susceptibility genes and that genetic and functional data sets can be merged to maximize discovery efforts. We propose that low *CDKN1B* levels seem to be involved in neuroblastoma initiation/progression and the T allele in promoter of *CDKN1B* is associated with low mRNA levels and confers the risk for neuroblastoma development.

## Conflict of interest

The authors confirm that there is no conflict of interest.

## Supporting information


**Data S1** Supplementary data.
