# Supplementary material for: The functional variant rs34330 of CDKN1B is associated with risk of neuroblastoma
Source: J Cell Mol Med. 2017 Jun 30;21(12):3224–30. doi: 10.1111/jcmm.13226 (PMC5706517; doi:10.1111/jcmm.13226)
Supplement: Supplementary file 1 — Data S1 Supplementary data. [file JCMM-21-3224-s001.doc]

**SUPPLEMENTARY INFORMATION**

**The functional variant rs34330 of *CDKN1B* is associated with risk of neuroblastoma**

*Mario Capasso ^1,2^, *Lee D. McDaniel^3,4^, Flora Cimmino^1,5^, Andrea Cirino^1,5^, Daniela Formicola^1,5^, Mike R. Russell^3,4^, Pichai Rahman^3,4^, Kristina A. Cole^3,4,6^, Sharon J. Diskin^3,4,6,7^.

^1^Università degli Studi di Napoli Federico II, Dipartimento di Medicina Molecolare e Biotecnologie Mediche, via Sergio Pansini 5, 80131 Naples, Italy.

^2^IRCCS SDN, Istituto di Ricerca Diagnostica e Nucleare, Via Gianturco 113, 80143 Naples, Italy

^3^Division of Oncology, Children’s Hospital of Philadelphia, 34th St. and Civic Center Blvd., Philadelphia, PA 19104-4399, USA

^4^Center for Childhood Cancer Research, Children’s Hospital of Philadelphia, 34th St. and Civic Center Blvd., Philadelphia, PA 19104-4399, USA

^5^CEINGE Biotecnolgie Avanzate, via Gaetano Salvatore 486, 80145 Naples, Italy

^6^Department of Pediatrics, Perelman School of Medicine, University of Pennsylvania, 3400 Civic Center Blvd, Philadelphia, PA 19104, USA

^7^Abramson Family Cancer Research Institute, Perelman School of Medicine at the University of Pennsylvania, 421 Curie Blvd, Philadelphia, PA 19104, USA

*These authors contributed equally to this work

**Corresponding Author:** Mario Capasso

Dipartimento di Medicina Molecolare e Biotecnologie Mediche, Università degli Studi di Napoli Federico II, CEINGE Biotecnologie Avanzate, Via G. Salvatore 486, Naples 80145, Italy. Tel: +390813737889; Fax: +390813737804, Email: mario.capasso@unina.it

**Patients and control subjects**

***Discovery Cohort of European Ancestry.*** Case subjects were defined as children affected by neuroblastoma or ganglioneuroblastoma and registered through the Children’s Oncology Group. The blood samples from the patients with neuroblastoma were recognized through the neuroblastoma biorepository of Children’s Oncology Group for specimen collection at the time of diagnosis. The suitability criterion for genome-wide genotyping was the availability of 1.5 μg of DNA of high quality from a tumor-free source such as peripheral blood or bone marrow mononuclear cells, which were not involved with a tumor. Control subjects were recruited from the Philadelphia region through the Children’s Hospital of Philadelphia Health Care Network, including four primary care clinics and different group practices and outpatient practices, which included well-child visits. Eligibility criteria for control subjects were European ancestry as determined by self-report or parental report, availability of 1.5 μg of high-quality DNA from peripheral-blood mononuclear cells, and no serious underlying medical disorder, obviously, including cancer.

***Italian Replication Cohort.*** Case subjects were defined as children with a diagnosis of neuroblastoma or ganglioneuroblastoma and collected through the Italian Neuroblastoma Group. Also in this case, the eligibility criterion for genotyping was the availability of DNA. All control subjects were recruited from Italian blood donor centers and the minimum requests were Italian origin, availability of DNA and no serious underlying medical disorder, including cancer. Main clinical and biological characteristics of the patients, including age, stage of the disease (INSS – International Neuroblastoma Staging System), and *MYCN* amplification status, are shown in the table below. Samples were assigned into two risk groups (not high-risk and high-risk) based on the COG (Children’s Oncology Group) risk assignment .

Neuroblastoma patient characteristics

|  | **Discovery Cohort of European Ancestry** |  | **Italian**  **Replication Cohort** |
| --- | --- | --- | --- |
|  | N=2101 |  | N=311 |
| Variable | Number (%) |  | Number (%) |
| **Age** |  | |  |
| >=18m | *736 (36) | | 142 (47) |
| <18m | 1324 (64) | | 163 (53) |
| Unknown | 41 | | 6 |
| **INSS Stage** |  |  |  |
| 4 | 875 (44) | | 129 (43) |
| 1,2,3,4s | 1110 (56) | | 174 (57) |
| Unknown | 116 | | 8 |
| **MYCN** |  | |  |
| Amplified | 342 (18) | | 74 (25) |
| Not amplified | 1566 (82) | | 219 (75) |
| Unknown | 193 | | 18 |
| **Risk** |  | |  |
| High-risk | 853 (44) | | 135 (43) |
| Not High-risk | 1091 (56) | | 176 (57) |
| Unknown | 157 | | - |
| *Divided using as cut off age=1 | |  |  |

**Genome-wide association study of neuroblastoma samples**

***Genome-wide SNP genotyping.*** Genotyping for discovery phase (European Americans) was performed using the Illumina Infinium II HumanHap550 and Human Quad610 Bead Chips according to methods detailed elsewhere and summarized here. DNA samples were surveyed for quality, both by optical density spectrophotometry and PicoGreen assay (Invitrogen). A total of 750 ng of DNA isolated from blood was used to genotype each sample according to the manufacturer’s guide. On day 1, genomic DNA was amplified 1.000-1.500-fold. On day 2, amplified DNA was fragmented to ~300-600 bp in size and was precipitated, re-suspended and then hybridized to the Bead Chip arrays. Single-base extension (SBE) used a single probe sequence to approximately 50 base pair designed to hybridize immediately adjacent to SNP query site. Following targeted hybridization to the array, locus-specifics primers for the arrayed SNP (attached to the beads) were extended with a single hapten-labeled dideoxynucleotide in the SBE reaction. The haptens were detected by a multilayer immunohistochemical sandwich assay. The Illumina Bead Array Reader scanned each Bead Chip at two wavelength and produced an image file. As Bead Chip images were collected, intensity values collected,which were determined for all instances of each bead type, and data files were created that summarized intensity values for each bead type. These files consisted of intensity data, which were loaded directly into the Illumina Genome Studio genotype analysis software. After normalization, the clustering algorithm was run to evaluate cluster positions for each locus and assign individual genotypes. From all genotypes, only samples with a genotype call rate ≥95% were considered for inclusion in this study.

***Quality control and association testing for the discovery cohort.*** Overlap of the HumanHAp550 v1, HumanHap550 v3 and Quad610 arrays. Our analysis only considered markers shared by HumanHap550 v1, HumanHap550 v3 and Quad610 arrays. Overall, 535,752 markers are shared by all three arrays and therefore were considered in this study.

***Low genotype call rate (<95%).*** Call rate was calculated on the basis of the number of “no call” genotypes, with default genotyping calling algorithm implemented in Illumina Genome Studio software. We did not consider any sample with a call rate of <95% for inclusion in this study. The call rate per individual was assessed by PLINK software (http://pngu.mgh.harvard.edu/~purcell/plink/) and confirmed to exceed 95% for all individuals, with an average genotyping rate of 99.85% across included individuals.

***SNP genotyping in Italian cohort.*** The SNP rs34330 was genotyped by Assay on 7900HT Real-time PCR system (Applied Biosystem). To monitor quality control, three DNA samples per genotype were genotyped by Sanger sequencing (3730 DNA analyzer, Applied Biosystem) and included in each 384-well reaction plate; genotype concordance was 100%. To confirm genotypes, we sequenced 20 samples chosen randomly from cases and controls; concordance between genotype was 100%. Primer sequences are available upon demand.

**Genotype Imputation**

We performed genotype imputation at the *CDKN1B* locus in a discovery set of 2,101 cases and 4,202 controls of European ancestry. Pre-phasing was performed first using SHAPEIT . Rather than preselecting reference population, we elected to follow approach of Howie et al. and use a multi population reference panel with IMPUTE2 . Genotypes for markers located on chromosome 12 were extracted and mapped to the hg19 human reference genome using the LiftOver tool. Multi population haplotype data from the 1000 Genomes Project Phase 1 integrated release were downloaded from the 1000 Genome Project website. IMPUTE2 was applied with default parameters and Ne (effective population size in the population-genetic model) = 20,000. Following imputation, SNPs with MAF of <1% and/or IMPUTE2-info quality score of <0.8 were removed. The remaining SNPs were tested for association with neuroblastoma using the frequentist association test under the additive model implemented in SNPTEST .

***In Vitro* functional analysis**

***Construction of luciferase reporter gene plasmids.*** The 5’-UTR sequence of *CDKN1B* was cloned downstream of the luciferase gene. PCR primer contained recognition sites for restriction enzyme (Xbal) in the forward and the reverse primer were designed to amplify 5’UTR from the cDNA of a healthy subject homozygous for the rs34330-C and rs34330-T alleles. After cutting the fragment with Xbal we cloned it into the pGL3-TK vector (Promega). The resulting plasmid containing the rs34330-C and rs34330-T. Before cell transfection, the sequence of each construct was confirmed by direct sequencing.

***Transient transfection and luciferase reporter gene assay.*** HEK293T and SHEP-2 cells were transfected with pGl3-CDKN1B-CC or pGl3-CDKN1B-TT constructs, and were subsequently starved in serum-free medium for 8h. Cells were induced to re-enter the cell cycle by the addition of fresh medium supplemented with 10% FCS for 0, 12 and 24 hours. At these time points, the cells were harvested, lysed and analyzed for luciferase activity. Luciferase activity was normalized to the internal Renilla control, after subtraction of pGl3 Basic Vector activity. Data represent the means ± S.D. of three independent transfections.

***Statistical analysis.*** The significant difference between the different experiments was assessed by two-sided T-test.

**SNP-gene expression correlation analysis**

***Neuroblastoma Cell Lines.*** Gene expression for 20 neuroblastoma cell lines with available SNP genotyping was measured with the Affymetrix HuGene ST 1.0 array (GSE78061). The 'arrayQualityMetrics' package was used to check for batch effects and outliers and to ensure expression data was of appropriate quality. Data was then processed using the Robust Multi-array Average (RMA) normalization algorithm with a log2 transformation. QC and processing of gene expression data was performed within the R/Bioconductor framework. The significant difference of gene expression between genotype groups has been evaluated by Mann Whitney test.

***Neuroblastoma Tumors.*** We downloaded mRNA expression data from a highly annotated series of 130 prospectively collected diagnostic neuroblastoma primary tumors generated through the National Cancer Institute (NCI) TARGET project (phs000467; https://target.nci.nih.gov). A second publicly available mRNA expression data set that comprised 101 primary neuroblastoma was downloaded from GEO database (GSE3690). A subset (n=51) of these tumor samples has paired DNA samples (genotyped by SNP array) from whole blood, which were used in the genotype-expression association analysis. The expression measures for each probe set in the Affymetrix arrays was extracted and normalized using well-established Robust Multi-array Average (RMA) protocols from CEL files. The last probe set annotation file from Affimetrix’s website (http://www.affymetrix.com) was used to assign expression value for each probe set to the corresponding gene. Statistical significance was evaluated by Mann Whitney test.

**References**

1. **Breslow N, McCann B.** Statistical estimation of prognosis for children with neuroblastoma. *Cancer Res*. 1971; 31: 2098-103.

2. **Capasso M, Devoto M, Hou C, Asgharzadeh S, Glessner JT, Attiyeh EF, Mosse YP, Kim C, Diskin SJ, Cole KA, Bosse K, Diamond M, Laudenslager M, Winter C, Bradfield JP, Scott RH, Jagannathan J, Garris M, McConville C, London WB, Seeger RC, Grant SF, Li H, Rahman N, Rappaport E, Hakonarson H, Maris JM.** Common variations in BARD1 influence susceptibility to high-risk neuroblastoma. *Nat Genet*. 2009; 41: 718-23.

3. **Steemers FJ, Chang W, Lee G, Barker DL, Shen R, Gunderson KL.** Whole-genome genotyping with the single-base extension assay. *Nat Methods*. 2006; 3: 31-3.

4. **Delaneau O, Zagury JF, Marchini J.** Improved whole-chromosome phasing for disease and population genetic studies. *Nat Methods*. 2013; 10: 5-6.

5. **Howie BN, Donnelly P, Marchini J.** A flexible and accurate genotype imputation method for the next generation of genome-wide association studies. *PLoS Genet*. 2009; 5: e1000529.

6. **Marchini J, Howie B, Myers S, McVean G, Donnelly P.** A new multipoint method for genome-wide association studies by imputation of genotypes. *Nat Genet*. 2007; 39: 906-13.

7. **Kauffmann A, Gentleman R, Huber W.** arrayQualityMetrics--a bioconductor package for quality assessment of microarray data. *Bioinformatics*. 2009; 25: 415-6.

8. **Irizarry RA, Hobbs B, Collin F, Beazer-Barclay YD, Antonellis KJ, Scherf U, Speed TP.** Exploration, normalization, and summaries of high density oligonucleotide array probe level data. *Biostatistics*. 2003; 4: 249-64.

9. **Wang Q, Diskin S, Rappaport E, Attiyeh E, Mosse Y, Shue D, Seiser E, Jagannathan J, Shusterman S, Bansal M, Khazi D, Winter C, Okawa E, Grant G, Cnaan A, Zhao H, Cheung NK, Gerald W, London W, Matthay KK, Brodeur GM, Maris JM.** Integrative genomics identifies distinct molecular classes of neuroblastoma and shows that multiple genes are targeted by regional alterations in DNA copy number. *Cancer Res*. 2006; 66: 6050-62.
